# Advances in β-Thalassemia Gene Therapy: CRISPR/Cas Systems and Delivery Innovations

**DOI:** 10.3390/cells14201595

**Published:** 2025-10-14

**Authors:** Hongmei Liu, Peng Zhang

**Affiliations:** 1 Center for Tissue Engineering and Stem Cell Research, Key Laboratory of Functional Nucleic Acids-Based Biopharmaceutical Research, Guizhou Biomanufacturing Laboratory, Guizhou Medical University, Guiyang 550031, China; lhm2017851002@163.com; 2 CAS Key Laboratory of Regenerative Biology, Guangzhou Institutes of Biomedicine and Health, Chinese Academy of Sciences, Guangzhou 510530, China

**Keywords:** β-thalassemia, CRISPR gene editing, fetal hemoglobin induction, delivery systems, hematopoietic stem cells

## Abstract

β-thalassemia is an inherited blood disorder caused by mutations in the β-globin (HBB) gene, leading to reduced or absent β-globin production, resulting in chronic anemia. While current therapies, including blood transfusions and hematopoietic stem cell transplantation, offer symptomatic relief, they are limited by complications and their limited accessibility. CRISPR-based gene editing technologies provide new therapeutic avenues by enabling the precise correction of HBB mutations or the reactivation of fetal hemoglobin (HbF) through the targeting of regulatory elements such as BCL11A. These approaches have shown promising preclinical and clinical outcomes. However, efficient and safe delivery remains a major challenge. Viral vectors offer high efficiency but raise concerns about immunogenicity and insertional mutagenesis, whereas non-viral systems such as lipid nanoparticles and engineered exosomes offer lower toxicity and modularity but face targeting limitations. This review highlights recent progress in CRISPR-based therapies for β-thalassemia and emerging delivery strategies to enhance clinical translation.

## 1. Introduction

β-thalassemia is a hereditary hematological disorder caused by mutations in the β-globin gene (HBB), leading to deficient or absent production of β-globin chains [1]. This imbalance results in ineffective erythropoiesis, hemolysis, and anemia of varying degrees of severity, significantly impacting patients’ quality of life [2]. Current clinical management primarily includes regular blood transfusions coupled with iron chelation therapy to manage iron overload, yet these treatments are associated with complications such as transfusion dependency, organ toxicity, and a substantial healthcare burden [3]. Although hematopoietic stem cell transplantation offers a potential cure, its use remains limited due to uncertain donor availability, transplant-related morbidity, and immunological incompatibility.

Recent advancements in gene editing technologies, particularly CRISPR-based platforms including CRISPR/Cas9, base editors (BEs), and prime editors (PEs), have introduced transformative therapeutic approaches in β-thalassemia [4]. These tools enable precise genetic modifications to correct disease-causing mutations, restore β-globin expression, or reactivate fetal hemoglobin (HbF) production through targeted epigenetic modulation, significantly alleviating clinical manifestations. CRISPR/Cas9 systems have proven effective in creating precise double-stranded breaks (DSBs) for gene editing, while base editors and prime editors offer additional advantages by enabling direct nucleotide conversions or targeted small insertions and deletions without inducing double-stranded breaks [5]. These approaches significantly alleviate clinical manifestations by addressing the underlying genetic defects rather than merely mitigating symptoms.

However, the clinical translation of these gene editing technologies critically depends on efficient, safe, and targeted delivery systems. Viral vectors such as lentiviruses and adeno-associated viruses (AAVs) have demonstrated substantial efficacy but raise concerns related to immunogenicity, integration risk, and limited packaging capacity [6]. Non-viral alternatives, including lipid nanoparticles (LNPs) and engineered exosomes, provide promising avenues due to their customizable properties, reduced immunogenic profiles, and potential for improved tissue specificity and controlled release [7]. Despite these advancements, each delivery approach has distinct limitations and requires careful optimization to maximize therapeutic potential while ensuring safety and efficacy.

This review comprehensively examines recent progress in CRISPR-mediated gene editing for β-thalassemia, emphasizing various delivery methodologies and their comparative advantages and limitations, and outlining critical challenges and future perspectives for personalized and clinically viable gene editing strategies. In contrast with prior reviews that primarily focused on summarizing editing strategies or clinical outcomes, our work provides a critical comparative synthesis of emerging CRISPR modalities, including base editing, prime editing, and CRISPRa/i, and integrates this into an in-depth discussion of delivery innovations, which are rapidly reshaping translational feasibility. Moreover, we highlight key regulatory, manufacturing, and cost considerations that are often overlooked but essential in bridging preclinical advances and clinical implementation. Together, these perspectives aim to guide future efforts to achieve the safe, effective, and scalable application of genome editing in β-thalassemia.

## 2. Molecular Basis and Clinical Phenotypes of Beta-Thalassemia

Thalassemia syndromes are caused by inherited defects in the genes encoding α- or β-globin chains, leading to imbalanced globin synthesis and ineffective erythropoiesis [8]. The α-globin genes (HBA1 and HBA2) reside on chromosome 16p13.3 [9], while HBB is located on chromosome 11p15.5 [1], within a locus that includes other globin genes expressed during embryonic and fetal development [2,10].

α-thalassemia is most commonly associated with large deletions involving one or both α-globin genes, such as --THAI, --SEA, --FIL, --MED, -α20.5, -α3.7, and –α4.2 [9]. In contrast, β-thalassemia results predominantly from point mutations in the HBB gene [1]. Based on residual β-globin output, genotypes are categorized as β^0^ (absent expression) or β^+^ (reduced expression), which are correlated with clinical severity [11]. A computational study analyzing HBB variation across databases (ClinVar, dbSNP, HbVar, and the 1000 Genomes Project) revealed 950 HBB variants, including 118 β^0^ and 48 β^+^ mutations [12]. Notably, intronic mutations such as IVS2-654 C > T (HBB: c.316-197C > T) and IVS1-110 G > A (HBB: c.93-21G > A) can generate aberrant splice sites, resulting in mis-spliced mRNA and impaired β-globin protein production [13,14,15].

**Figure 1:**
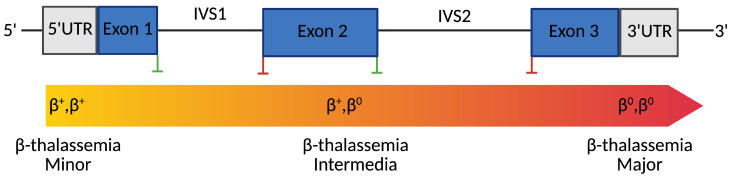
Genotype-Phenotype Correlation in β-Thalassemia. Created with Bio-Render.

Clinically, β-thalassemia encompasses a spectrum of phenotypes classified into β-thalassemia minor, intermedia, and major [16]. However, emerging molecular and biochemical insights increasingly reveal substantial intragroup variability. β-thalassemia major, or transfusion-dependent thalassemia (TDT), represents the most severe form [17]. Within the first two years of life, affected individuals typically present with profound anemia, failure to thrive, hepatosplenomegaly, and bone changes due to marrow expansion. These patients are transfusion-dependent throughout their life and are at risk of complications such as iron overload, cardiac dysfunction, and endocrine abnormalities if not properly managed [18,19]. β-thalassemia intermedia refers to an intermediate phenotype [17]. Patients exhibit moderate anemia and may not require regular transfusions. This condition often arises from compound heterozygosity for mild and severe β-thalassemia mutations or the co-inheritance of genetic modifiers such as α-thalassemia deletions or polymorphisms that elevate HbF levels (e.g., in BCL11A, KLF1). Clinical manifestations vary and may include splenomegaly, extramedullary hematopoiesis, bone deformities, and iron overload due to increased intestinal absorption [20,21]. β-thalassemia minor is typically asymptomatic and detected incidentally during routine blood testing [17]. It results from heterozygous mutations in the HBB gene and is characterized by microcytic, hypochromic red blood cells with mild anemia [22,23,24] (Figure 1).

Schematic representation of the human β-globin (HBB) gene structure and its correlation with clinical phenotypes. Blue boxes indicate exons; gray boxes represent untranslated regions (UTRs). Green vertical lines mark splice donor sites, while red lines indicate splice acceptor sites. The color gradient bar below the gene illustrates the clinical phenotype continuum associated with different β-globin genotypes, from β^+^/β^+^ (minor), through β^+^/β^0^ (intermediate), to β^0^/β^0^ (major). Mutation types distributed along the gene, including promoter mutations, splice-site variants, nonsense variants, and frameshift mutations, determine the degree of residual β-globin expression, ultimately influencing disease severity.

## 3. CRISPR-Based Genome Editing for β-Thalassemia

**Figure 2:**
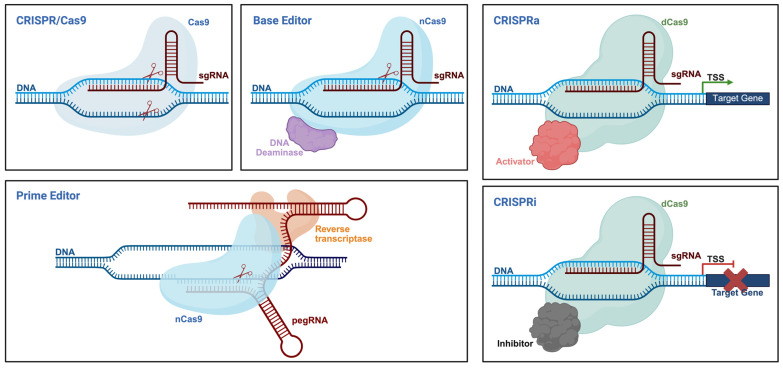
Schematic overview of CRISPR-based gene editing platforms. Created with Bio-Render.

The emergence of gene editing technologies has opened up powerful therapeutic avenues for the treatment of β-thalassemia. By enabling the precise manipulation of disease-causing genes or their regulatory elements, these tools offer the potential for durable, one-time genetic correction. As illustrated in Figure 2, CRISPR-based technologies have evolved into a diverse and modular toolkit. The CRISPR/Cas9 system enables site-specific DNA cleavage and gene disruption. Base editors (BEs) allow precise single-nucleotide conversions without introducing double-strand breaks, while prime editors (PEs) enable a broader range of edits, including insertions and deletions, with high precision. Additionally, catalytically inactive Cas9 (dCas9) fused to transcriptional regulators can modulate gene expression without altering the underlying DNA sequence (CRISPRa/i). Together, these platforms provide a versatile foundation for the development of targeted, gene-based therapies.

The following provides a comparison of major CRISPR-derived technologies used for genome and epigenome engineering.

### 3.1. CRISPR/Cas9, Base Editors, and Prime Editors

The CRISPR/Cas9 system, originally discovered as a bacterial adaptive immune mechanism, has emerged as a powerful and programmable platform for genome editing [25]. Its core mechanism involves a single guide RNA (gRNA) that directs the Cas9 endonuclease to a specific genomic locus, where it introduces DSBs [25]. This break is subsequently repaired by the cell’s endogenous DNA repair pathways, primarily non-homologous end joining (NHEJ), homology-directed repair (HDR), and microhomology-mediated end-joining (MMEJ) [26]. Since its first application in eukaryotic systems in 2013, CRISPR/Cas9 has undergone substantial refinement, leading to the development of next-generation editing tools such as BEs and PEs [27]. Base editors, such as adenine base editor (ABEs) and cytosine base editor (CBEs), are engineered by fusing Cas9 nickase (nCas9) with DNA deaminases (e.g., TadA for ABE, APOBEC for CBE), allowing efficient A•T to G•C or C•G to T•A conversions within a defined editing window, without generating DSBs [28,29]. PE, representing a more versatile and precise genome editing modality, consists of an nCas9 fused to a reverse transcriptase (RT), and utilizes a specialized prime editing guide RNA (pegRNA) that includes a primer binding site (PBS) and a reverse transcription template (RTT) [30]. This configuration enables targeted insertions, deletions, and all 12 types of point mutations, without requiring exogenous donor DNA or inducing DSBs [30]. Given their precision, versatility, and expanding editing capabilities, CRISPR-based technologies have opened up promising new avenues for the treatment of monogenic disorders, including β-thalassemia. Compared to earlier genome engineering tools such as zinc finger nucleases (ZFNs) and transcription activator-like effector nucleases (TALENs), CRISPR-based tools enable easier and more efficient targeting of endogenous genomic loci [31]. This facilitates the correction of pathogenic mutations or the modulation of regulatory elements, yielding therapeutic outcomes that are both durable and physiologically relevant.

### 3.2. dCas9-Based Transcriptional and Epigenetic Modulation

Beyond direct sequence modifications, recent advancements in gene editing technologies have enabled the precise modulation of gene expression through epigenetic mechanisms, offering therapeutic potential without altering the underlying DNA sequence. One such approach involves CRISPR activation (CRISPRa) [32] and CRISPR interference (CRISPRi) systems [33], which utilize catalytically dead Cas9 (dCas9) fused to transcriptional effectors [34]. CRISPRa systems enable the precise and reversible upregulation of endogenous gene expression without altering the underlying DNA sequence. These systems typically rely on dCas9 fused to transcriptional activators such as VP64, p65, or Rta [35,36,37]. Upon targeting promoter or enhancer regions via gRNAs, the fusion complex recruits the transcriptional machinery to enhance gene expression [38]. To overcome the limitations of conventional CRISPRa systems, particularly the requirement for multiple gRNAs to achieve strong activation, more advanced architectures have been developed, including SunTag, VPN, and synergistic activation mediator (SAM) systems. The SunTag platform employs a multimeric peptide scaffold that recruits multiple VP64 copies to a single dCas9, thereby amplifying the transcriptional output from a single target site [39]. The VPN system integrates VP48, p65, and human heat-shock factor 1 (HSF1) into a single tripartite fusion domain, significantly enhancing transcriptional activation at compact loci [40]. The SAM system further boosts gene activation by engineering gRNAs to contain MS2 RNA aptamers, which in turn recruit additional transcriptional co-activators such as p65 and HSF1 via MS2-binding fusion proteins [41]. Conversely, CRISPRi is a programmable and reversible gene silencing strategy. By guiding dCas9 to promoter regions or near transcriptional start sites (TSSs) using specific single-guide RNAs (sgRNAs), CRISPRi sterically blocks RNA polymerase binding or elongation, thereby reducing gene expression [42]. Gene repression efficiency can be significantly enhanced by fusing dCas9 to transcriptional repressors such as KRAB (Krüppel-associated box), which recruits epigenetic modifiers to establish repressive chromatin states [43,44]. Variants such as dCas9-KRAB-MeCP2 and dCas9-KRAB-ZIM3 have demonstrated improved silencing efficiency, especially for long-term repression [45,46]. In addition to transcriptional activators and repressors, dCas9 can also be fused to epigenetic effectors to directly modulate chromatin states. For example, dCas9-LSD1 has been used to remove histone methylation marks (H3K4me1/2) at enhancers, leading to targeted gene repression in a locus- and cell-type-specific manner [47]. Similarly, the fusion of dCas9 to the catalytic core of the acetyltransferase p300 enables localized H3K27 acetylation and activation of gene expression without inducing double-stranded breaks [48]. These epigenetic editing tools expand the CRISPR toolbox by enabling heritable yet reversible transcriptional control, offering unique advantages in interrogating regulatory elements and fine-tuning disease-associated gene networks.

## 4. CRISPR-Mediated Correction Strategies for β-Thalassemia

Significant progress has been made in applying CRISPR-based editing strategies to β-thalassemia, particularly through the direct correction of HBB mutations or the modulation of HbF expression via HBG gene regulation. Early efforts focused on gene correction in patient-derived induced pluripotent stem cells (iPSCs). In 2015, the pathogenic HBB: c.52A > T mutation was successfully corrected with 16.67% efficiency using CRISPR/Cas9, leading to restored HBB gene expression in edited iPSCs [49]. Subsequent studies achieved up to 20% correction of the IVS2-654 (C > T) mutation in iPSCs [50,51], and the efficient repair of the common frameshift mutation HBB: c.126-129delCTTT was also demonstrated by multiple groups [52,53,54]. These early successes in iPSCs laid the foundation for translational studies in hematopoietic stem and progenitor cells (HSPCs), which represent a more clinically relevant target for autologous cell therapy. In a 2020 in vivo study, CRISPR/Cas9 components (sgRNA and Cas9 mRNA) were microinjected into single-cell mouse embryos carrying the IVS-2-654 C > T mutation. Remarkably, 70% of the live-born mice exhibited the correction of RNA splicing and restored hematologic parameters and tissue morphology, demonstrating a durable and systemic therapeutic effect [55]. More recently, the development of base editing technologies has offered a safer and more efficient alternative for correcting point mutations without introducing double-strand breaks. In 2023, Hardouin et al. used adenine base editors (ABEs) delivered via RNA to correct the common IVS1-110 (G > A) mutation in patient-derived HSPCs, achieving ~80% correction with minimal toxicity and preserving stem cell function [56]. In parallel, Badat et al. applied an optimized base editor, ABE8e, to efficiently revert the HbE mutation in patient-derived HSPCs, with up to 90% editing efficiency and the complete restoration of adult hemoglobin expression [57]. Prime editing (PE3) successfully corrected multiple clinically relevant mutations (CD6, CD17, CD19, IVS-I-1, etc.) in human erythroid precursor cells (HUDEP-2), with efficiencies ranging from approximately 10% to 50%, achieving precise editing without detectable off-target effects [58].

Beyond the direct correction of HBB mutations, the reactivation of fetal γ-globin expression through the disruption of the BCL11A regulatory pathway has emerged as one of the most effective therapeutic strategies for β-thalassemia. BCL11A is a zinc finger transcription factor that plays a pivotal role in the developmental switch from fetal (γ-globin) to adult (β-globin) hemoglobin. During fetal development, γ-globin expression is high, producing fetal hemoglobin (HbF, α_2_γ_2_), which can compensate for β-globin deficiency and ameliorate disease severity in β-hemoglobinopathies [59]. After birth, BCL11A binds to the promoters of HBG1 and HBG2 and recruits chromatin remodeling complexes that silence γ-globin transcription, leading to the switch to adult hemoglobin [60]. Genome-wide association studies have identified single-nucleotide polymorphisms within the erythroid-specific enhancer of BCL11A that correlate with persistently high HbF levels and milder disease phenotypes, highlighting this enhancer as a critical therapeutic target.

One of the most effective approaches involves disrupting the erythroid-specific enhancer of BCL11A in CD34^+^ HSPCs using CRISPR/Cas9 editing. This enhancer-targeting strategy leads to the selective silencing of BCL11A in erythroid cells, sparing its function in other tissues. The clinical application of this strategy has shown remarkable results: patients with transfusion-dependent β-thalassemia who received edited autologous HSPCs demonstrated a sustained induction of HbF and achieved transfusion independence [61,62]. Comparative genome editing studies further highlighted the superiority of BCL11A enhancer editing over other targets such as KLF1 and HBG1/2, showing robust HbF reactivation with minimal off-target effects or transcriptional disturbances [63]. Refinements in mechanistic understanding have enabled more precise targeting strategies. Rajendiran et al. employed base editing to selectively modify key zinc finger (ZnF) domains in the erythroid-specific BCL11A-XL isoform. Editing ZnF4 alone was sufficient to relieve γ-globin repression while preserving normal hematopoiesis, whereas edits at ZnF5/6 induced broader effects on erythroid function [64]. In parallel, Han et al. applied a cytosine “transformer” base editor (tBE) to directly disrupt BCL11A-binding motifs in the HBG1/2 promoters within human CD34^+^ HSPCs. This approach achieved potent and durable HbF upregulation, surpassing traditional ABE8e or Cas9 nuclease editing, with undetectable off-target edits and long-term stability in repopulating stem cells [65]. Complementary strategies also target the HBG promoter directly. Traxler et al. designed CRISPR/Cas9 constructs to edit a 13-nt sequence (−102 to −114) within the HBG1 promoter, a region deleted in hereditary persistence of HbF. Editing this motif in HUDEP-2 cells and CD34^+^ HSPCs increased HbF levels up to 46%, reversed the γ-to-β switch, and maintained normal erythroid differentiation. Moreover, in patient-derived cells from individuals with sickle cell disease, this edit reduced hypoxia-induced sickling, highlighting its translational relevance [66].

Collectively, these studies reveal that CRISPR-based editing strategies are complementary yet fundamentally different solutions for β-thalassemia therapy. Each platform brings unique strengths as well as inherent limitations that must be critically considered when exploring clinical translation (Table 1). Traditional CRISPR/Cas9 remains a powerful tool for disrupting regulatory elements and correcting frameshift mutations, but its reliance on double-strand breaks raises concerns about off-target cleavage, genotoxicity, and p53 activation, particularly in long-term repopulating HSPCs. Base editors mitigate these risks by enabling precise single-base conversions without introducing double-strand breaks and have achieved high editing efficiencies in clinically relevant models. However, their dependence on PAM availability and the potential for bystander edits constrain their versatility and raise safety considerations regarding their clinical use. Prime editors further expand the editing landscape to include a wide range of mutation types, yet current challenges, such as lower efficiency, delivery complexity, and potential integration with large cargo packaging, must be overcome before they can be broadly applied in therapeutic settings. Importantly, mutation-agnostic strategies that reactivate fetal γ-globin expression through BCL11A enhancer disruption or promoter editing offer an alternative therapeutic paradigm, bypassing the need for patient-specific mutation correction and demonstrating robust, durable HbF induction with minimal off-target activity. These approaches hold particular promise for addressing genetic heterogeneity in β-thalassemia, but their long-term safety, hematopoietic specificity, and large-scale manufacturing possibilities remain to be determined. Together, these insights underscore that no single editing modality is universally optimal. Instead, therapeutic design must be tailored to the molecular nature of the mutation, the clinical context, and the logistical realities of gene therapy implementation. Addressing these challenges will be essential in transforming CRISPR-based genome editing from a powerful experimental tool into a widely applicable therapeutic platform for β-thalassemia and related hemoglobinopathies.

## 5. Emerging Gene Delivery Systems

The efficient and precise delivery of gene editing components represents a cornerstone in the clinical translation of CRISPR-based therapies. Delivery systems not only determine the efficacy of gene editing but also influence safety, targeting specificity, and feasibility for in vivo applications. Currently, commonly used delivery vectors include both viral and non-viral platforms.

### 5.1. Viral Vectors

Lentiviral vectors (LVs), derived from human immunodeficiency virus type 1 (HIV-1), are widely used for gene therapy in β-hemoglobinopathies, owing to their ability to efficiently transduce both dividing and non-dividing HSPCs and support stable, long-term transgene expression [67]. In β-thalassemia, two major LV-based strategies have been pursued: the reactivation of γ-globin through silencing of transcriptional repressors, and the addition of functional globin genes. The erythroid-specific knockdown of BCL11A via an LV-encoded microRNA-adapted shRNA (e.g., BCH-BB694) has been demonstrated to robustly induce HbF in both in vitro and in vivo settings without adverse effects on HSPC function, thus providing preclinical support for clinical translation in sickle cell disease and β-thalassemia [68]. Building on this, Liu et al. developed a lentiviral vector co-expressing shmiRs targeting both BCL11A and ZNF410, achieving even higher HbF induction and more effective correction of erythroid phenotypes in preclinical models of sickle cell disease and β-thalassemia [69]. In parallel, efforts to optimize β-globin gene delivery have focused on refining the regulatory elements within the vector. Morgan et al. employed high-resolution mapping (LV-MPRA) of the β-globin locus control region (LCR) to identify compact, potent enhancer elements, enabling the construction of novel LVs (e.g., CoreGA-AS3-FB) that showed improved viral titers and transduction efficiency while maintaining strong β-globin expression and phenotypic rescue in disease models [70,71]. Clinically, a recent single-arm pilot trial in Chinese pediatric patients with the most severe β^0^/β^0^-thalassemia genotype demonstrated that an insulator-engineered, β-globin-optimized LV product (BD211) enabled long-term transfusion independence and stable HbA^T87Q^ expression without notable adverse effects over nearly two years of follow-up, highlighting the therapeutic promise shown by this approach [72].

Adeno-associated virus (AAV) vectors are small, non-pathogenic, single-stranded DNA viruses that have become leading tools for in vivo gene delivery due to their broad tropism, high transduction efficiency, and favorable safety profile [73,74]. AAV serotype 6 (AAV6), in particular, is highly efficient in transducing HSPCs, making it an attractive platform for precise genome editing in β-hemoglobinopathies [75]. One promising approach involves the introduction of the naturally occurring hereditary persistence of HbF mutations into the promoters of HBG1/HBG2, thereby reactivating γ-globin expression and elevating HbF levels. Lu et al. showed that Cas9 RNPs combined with AAV6 donor templates could efficiently introduce six HPFH mutations into both HUDEP-2 cells and patient-derived HSPCs, leading to robust γ-globin induction and preserved multilineage engraftment in immunodeficient mice [76]. Another innovative strategy leverages AAV6 to mediate the replacement of the endogenous α-globin gene (HBA1) with a full-length β-globin (HBB) transgene in β-thalassemia-derived HSPCs. Cromer et al. demonstrated that this approach normalized β-globin: α-globin ratios, restored functional adult hemoglobin production, and supported long-term multilineage hematopoiesis following transplantation, offering a universal genome editing solution for β-thalassemia [77]. Additionally, AAV6-based gene correction has been applied to sickle cell disease (SCD) models. Wilkinson et al. reported that Cas9/AAV6 correction of the mutant HBB allele in autologous SCD mouse HSCs achieved stable engraftment, persistent hemoglobin-A production, and the correction of disease phenotypes after transplantation [78].

### 5.2. Non-Viral Approaches

Lipid nanoparticles (LNPs) are synthetic nanocarriers composed of ionizable lipids and helper lipids, engineered to encapsulate and deliver nucleic acids, including mRNA, siRNA, and gene editing tools, into cells with high efficiency and minimal immunogenicity [79,80,81]. Owing to their clinical success as mRNA vaccine platforms, LNPs have recently gained attention as next-generation vehicles for the in vivo delivery of gene editing cargos [82]. Recent studies have demonstrated that antibody-conjugated LNPs, targeting surface markers such as c-Kit (CD117), enable efficient and specific RNA delivery to HSPCs in vivo. Shi et al. developed an anti-CD117 antibody-targeted LNP system that, following a single intravenous injection, delivered Cre mRNA to nearly 90% of HSPCs and long-term HSCs (LT-HSCs) in mice. Edited cells retained their stemness and functionality, giving rise to high levels of gene-edited mature immune cells over time. This proof of concept established that LNPs can efficiently access the bone marrow niche and stably edit HSCs and their progeny in situ, thus providing a potential path toward in vivo genetic therapies for hematological disorders [83]. In a complementary approach, Breda et al. reported that antibody-targeted LNPs could be used not only for efficient mRNA delivery and genome editing in murine and human HSCs but also for delivering pro-apoptotic mRNA cargos to selectively deplete HSCs in vivo, enabling non-genotoxic conditioning regimens for transplantation [84].

Exosomes are nanosized extracellular vesicles naturally secreted by nearly all cell types, playing essential roles in intercellular communication by transferring proteins, nucleic acids, and lipids to recipient cells [85]. Engineered exosomes are artificially modified or loaded with specific cargos (such as nucleic acids or proteins) and/or surface ligands to enhance their targeting ability and therapeutic potential [86]. Due to their innate biocompatibility, low immunogenicity, and capability to cross biological barriers, exosomes are increasingly recognized as promising vehicles for targeted drug and gene delivery [87,88,89]. Recent studies have demonstrated that exosomes, especially when engineered, can serve as highly effective, non-viral delivery systems for nucleic acid-based therapeutics and gene editing tools, enabling precise and safe genome modification in vivo. For example, Li et al. developed an exosome-based platform for mRNA delivery by encapsulating Ldlr mRNA into exosomes derived from liver cells. The systemic administration of these exosomes in an Ldlr-deficient mouse model resulted in efficient uptake by hepatocytes and immune cells, the robust restoration of LDLR protein expression, and significant improvement to hypercholesterolemia and atherosclerosis phenotypes, illustrating the potential of exosome-mediated mRNA therapy in inherited metabolic diseases [90]. Expanding the application to gene editing, Wan et al. established a method for the exosome-mediated delivery of Cas9 ribonucleoprotein (RNP) complexes via electroporation into purified exosomes from hepatic stellate cells. These exosome–RNP complexes achieved efficient, tissue-specific genome editing in liver tissue after systemic injection, leading to therapeutic effects in multiple disease models, including acute liver injury, chronic fibrosis, and hepatocellular carcinoma, by targeting relevant disease genes. This study also confirmed the biocompatibility, low immunogenicity, and stability of exosome-RNP complexes in vivo, supporting their translational potential in genome editing-based therapies [91].

Different delivery systems for gene editing, including viral vectors, lipid nanoparticles, and engineered exosomes, each possess distinct advantages and inherent limitations (Table 2). Viral vectors such as lentivirus and AAV offer high transduction efficiency and stable gene expression, and their clinical utility is supported by numerous successful trials. Among current options, viral platforms remain the closest to clinical readiness; however, significant hurdles persist, including the risks of insertional mutagenesis, pre-existing immunity, restricted cargo capacity, particularly for large editors such as PEs, and the complexity and cost of large-scale GMP manufacturing. Non-viral systems such as LNPs provide modularity, reduced immunogenicity, and transient expression, making them attractive for in vivo editing and applications where permanent genomic integration is undesirable. Importantly, antibody- or peptide-modified LNPs have shown improved hematopoietic specificity and enabled targeted delivery to CD34^+^ HSPCs in preclinical models. Yet, despite this progress, most LNP-based approaches remain at the proof-of-concept stage. Their clinical translation is limited by inefficient endosomal escape, suboptimal bone marrow homing, dose-dependent toxicity, and the absence of robust, scalable manufacturing pipelines. Engineered exosomes represent an emerging frontier with unique advantages in biocompatibility, immune evasion, and the capacity to cross biological barriers. While promising results in animal models underscore their potential for precise in vivo genome editing, exosome-based systems are still at an early stage of development. Key obstacles, including low cargo loading efficiency, variability in targeting specificity, and challenges in standardized large-scale production, must be addressed before clinical deployment can be achieved.

Overall, a more critical perspective highlights that while viral vectors currently lead the field in clinical maturity, non-viral systems hold greater long-term potential for safer, repeatable, and potentially in vivo–applicable therapies. Achieving this transition will require innovations in targeted delivery to hematopoietic niches, improved efficiency in intracellular trafficking, scalable manufacturing, and comprehensive safety assessments. At the same time, lessons learned from CRISPR applications in other hematologic and solid tumors may inform β-thalassemia therapy. For example, CRISPR-based liquid biopsy assays have demonstrated the ultra-sensitive detection of rare NRAS mutant ctDNA in melanoma patients, providing a framework for monitoring editing outcomes and off-target events in gene-edited HSPCs [92]. Similarly, exosomes are increasingly being recognized as not only delivery vehicles but also active mediators of intercellular communication and therapy resistance. A recent study showed that EV miRNA cargo contributes to chemoresistance in breast cancer, underscoring the need to consider potential functional consequences of edited cell-derived exosomes in future therapeutic applications [93]. In summary, no single delivery platform is universally ideal, and the optimal choice will likely depend on the disease context, therapeutic objective, and translational feasibility.

## 6. Clinical Translation of CRISPR-Based Therapies in β-Thalassemia

The clinical translation of CRISPR-based genome editing in β-thalassemia has advanced from proof-of-concept studies to pivotal trials (Table 3), demonstrating remarkable therapeutic efficacy and a manageable safety profile. Among landmark clinical programs are the CLIMB THAL-111 and CLIMB SCD-121 trials, in which autologous CD34^+^ HSPCs were edited ex vivo in the erythroid-specific enhancer of BCL11A to reactivate HbF synthesis. The first in-human results, reported in 2021, showed that a single infusion of CTX001 led to sustained transfusion independence in a patient with transfusion-dependent β-thalassemia and the elimination of vaso-occlusive crises in a patient with sickle cell disease, with stable engraftment and no evidence of off-target editing over more than a year’s follow-up [62]. These pioneering results confirmed the clinical feasibility and safety of the CRISPR-mediated disruption of BCL11A for hemoglobinopathies.

Building on these findings, a multicenter phase 3 study further evaluated exagamglogene autotemcel (exa-cel) in a larger cohort (NCT03655678). In this trial, 52 patients aged 12–35 years received autologous CD34^+^ HSPCs edited at the BCL11A enhancer following myeloablative conditioning. Among the 35 patients with sufficient follow-up, 91% achieved durable transfusion independence, maintaining mean total hemoglobin levels of 13.1 g/dL and mean HbF levels of 11.9 g/dL, with pancellular distribution in over 94% of red cells. The safety profile was consistent with that of standard busulfan conditioning and autologous transplantation, with no treatment-related deaths or malignancies reported [94]. These results represent a major milestone, indicating that the targeted reactivation of fetal hemoglobin can functionally cure β-thalassemia in most treated patients. Parallel efforts in China have yielded similarly promising outcomes. In a clinical trial of BRL-101 (NCT04211480, NCT04205435), CRISPR-Cas9 editing of the BCL11A + 58 erythroid enhancer in pediatric patients led to transfusion independence in all six treated individuals, including those with the most severe β^0^/β^0^ genotypes. F-cell proportions exceeded 90% within six months, and the editing efficiency in peripheral blood mononuclear cells surpassed 60%. No treatment-related mortality occurred, and adverse events were generally attributable to conditioning and transplantation procedures; only transient thrombocytopenia was attributed to BRL-101 itself [95]. This trial highlights the reproducibility and global applicability of CRISPR-based strategies across different patient populations and clinical settings.

Beyond hematologic correction, CRISPR therapies have shown profound impacts on patient-reported outcomes. In an extended analysis of the CLIMB THAL-111 and CLIMB-131 studies, 54 patients followed for up to 48 months exhibited significant and sustained improvements in health-related quality of life (HRQoL) measures after exa-cel infusion. Adults reported clinically meaningful gains in EQ-5D-5L and FACT-BMT scores, while adolescents demonstrated substantial improvements in PedsQL scores, reflecting enhanced physical, emotional, and social well-being [96]. These results underscore the transformative potential of gene-edited autologous cell therapies not only in achieving transfusion independence but also in restoring quality of life.

## 7. Challenges and Future Perspectives

Although CRISPR-based therapies for β-thalassemia have entered clinical translation, current applications remain largely focused on the ex vivo editing of autologous HSPCs. This approach offers several advantages, including precise control of editing conditions, efficient screening for on-target activity and off-target safety before reinfusion, and consistently high editing efficiency, as observed in clinical trials. However, ex vivo editing requires myeloablative conditioning, specialized manufacturing infrastructure, and prolonged hospitalization, which limit its accessibility and scalability, especially in low-resource settings. In contrast, in vivo genome editing, in which CRISPR components are delivered directly to HSPCs within the patient, represents a highly promising future direction. By eliminating the need for conditioning and ex vivo manipulation, in vivo approaches could greatly simplify treatment and expand patient access. Nevertheless, major challenges remain.

A primary concern in clinical gene editing is the potential for off-target effects, which could lead to unintended genomic alterations and oncogenic risks. Precise targeting remains challenging due to sequence homology and the inherent activity of Cas enzymes [97]. Additionally, the variable editing efficiency, particularly observed in primary human HSPCs, limits the therapeutic potential [98]. To address these challenges, a number of optimization strategies have been developed. These include the engineering of high-fidelity Cas9 variants (e.g., eSpCas9, SpCas9-HF1, and HypaCas9) with reduced off-target activity [99], and the use of advanced guide RNA design algorithms that enhance on-target activity while minimizing off-target cleavage [100]. Beyond nuclease-based genome modification, transcriptional regulation platforms such as CRISPRi and CRISPRa represent a promising direction for future β-thalassemia therapies. CRISPRi can be leveraged to silence the expression of repressors such as BCL11A or LRF, thereby reactivating γ-globin and increasing HbF production. Conversely, CRISPRa systems enable the upregulation of endogenous HBG1/2 or HbF-promoting factors, mimicking the hereditary persistence of HbF.

The effective and targeted delivery of gene editing constructs remains a major challenge in translating genome editing technologies into clinical practice. Viral vectors, such as lentiviruses and adeno-associated viruses (AAVs), have demonstrated high transduction efficiency and stable gene expression. However, their use is accompanied by concerns regarding immunogenicity, insertional mutagenesis, and limited packaging capacity, particularly for larger constructs such as Cas9 and prime editors [101]. In contrast, non-viral delivery systems, including LNPs, EVs, and other physical methods, offer advantages such as lower immunogenicity, greater cargo versatility, and transient expression [102]. However, these systems often suffer from poor tissue penetration, inconsistent delivery to HSPCs, and limited targeting specificity. To overcome these limitations, recent studies have explored ligand-based surface modifications, such as antibody or aptamer conjugation, to direct delivery vehicles to specific cell types [103]. Engineered exosomes have also shown promise due to their membrane fusion capacity and modifiability, potentially enabling the delivery of Cas9 RNPs or mRNA directly into HSCs [91,104].

Beyond these scientific and technical considerations, several non-biological barriers must also be addressed before CRISPR therapies can achieve widespread clinical implementation. Regulatory challenges remain substantial: genome-edited cell products require extensive preclinical evaluation to determine their off-target activity, genotoxicity, and long-term safety, as well as robust quality control and reproducibility data to satisfy regulatory agency requirements. The absence of standardized guidelines for emerging modalities such as base and prime editing further complicates clinical approval pathways. Manufacturing complexity is another key limitation. Ex vivo gene editing involves individualized, GMP-compliant cell processing pipelines that are expensive, labor-intensive, and difficult to scale. Variability between production batches, limited vector yields, and supply chain constraints for key reagents lead to additional bottlenecks. Transitioning toward automated, closed-system platforms and improving the scalability of both viral and non-viral delivery systems will be critical in achieving broader clinical deployment. The high cost of gene editing therapies, often exceeding 1 million per patient, poses significant barriers to equitable access. These costs are driven by the complexity of autologous manufacturing, conditioning regimens, and long-term patient monitoring. Strategies such as in vivo editing, the development of allogeneic off-the-shelf products, and integration into public health reimbursement frameworks will be essential in enhancing affordability and expanding patient reach.

Collectively, these evolving strategies are designed to make gene editing not only more precise and efficient but also safely and effectively deliverable to clinically relevant target cells. However, addressing regulatory, manufacturing, and economic barriers will be just as critical as overcoming technical limitations. Only by combining scientific innovation with scalable production, regulatory clarity, and cost-effective implementation can we fully harness the transformative potential of CRISPR-based therapies for β-thalassemia and other monogenic hematological diseases.

## 8. Conclusions

CRISPR-based gene editing has emerged as a transformative therapeutic strategy for β-thalassemia, offering the potential for a one-time, durable correction to disease-causing mutations or the reactivation of HbF expression. In this review, we summarized recent advances in genome editing platforms, including Cas9 nucleases, BEs, PEs, and dCas9-based transcriptional regulators (CRISPRa/i). Furthermore, we highlighted the comparative strengths and limitations of emerging delivery systems, including lentiviral and AAV vectors, lipid nanoparticles, and engineered exosomes, with a focus on their applications in hematopoietic stem and progenitor cells. Despite substantial progress, challenges remain, including off-target effects, suboptimal editing efficiency in hematopoietic stem cells, and the need for safe, targeted delivery vehicles. This review also discussed future perspectives, such as leveraging CRISPRi/a for transcriptional modulation and refining delivery technologies for in vivo HSPC targeting. Continued interdisciplinary efforts to optimize editing precision, delivery platforms, and regulatory safety will be critical in harnessing the clinical potential of gene editing in β-thalassemia and other inherited blood disorders.

## Figures and Tables

**Table 1:** Comparative analysis of major CRISPR-based genome editing strategies for β-thalassemia.

Strategy	Editing Efficiency	Specificity	Advantages	Limitations	Translational Relevance
CRISPR/Cas9	High	Moderate	Robust for frameshift correction and enhancer disruption	Off-target cleavage, DSB-induced genotoxicity	Already in clinical trials
Base editing	Very high	High	Precise single-base changes without DSBs	PAM dependency, bystander edits	Preclinical models show high efficiency
Prime editing	Moderate	Very high	Versatile (all mutation types, small insertions/deletions)	Lower efficiency, delivery complexity	Promising but still preclinical
CRISPRa/i	N/A	Very high	Reversible, mutation-agnostic, no DNA breaks	Delivery and expression control	Potential for HbF induction

**Table 2:** Comparison of major packaging and delivery systems for CRISPR-based therapies.

Packaging System	Advantages	Limitations
LVs	Efficiently transduce dividing and non-dividing HSPCs; support stable, long-term gene expression; clinically validated	Risk of insertional mutagenesis; random integration; limited cargo size for large editors
AAV	High transduction efficiency; low immunogenicity; infects non-dividing cells	Small packaging capacity (~4.7 kb); pre-existing immunity; transient expression
LNPs	Low immunogenicity; transient expression avoids insertional risks; compatible with mRNA and RNP delivery	Potential consequences of non-uniform particle size; challenges with bone marrow homing; dose-dependent toxicity; endosomal escape inefficiency
Engineered exosomes	High biocompatibility; low immunogenicity; cross biological barriers; natural membrane fusion capacity	Low loading efficiency; limited targeting specificity; scalability challenges
Electroporation	Very high delivery; efficiency into CD34^+^; HSPCs; widely used in clinical trials	Not suitable for in vivo use; may cause cell toxicity

**Table 3:** Landmark clinical trials of CRISPR-based therapies for β-thalassemia.

Trial (NCT No.)	Target	Phase	Patients (n)	Key Efficacy Outcomes	Safety Outcomes	Ref.
CLIMB THAL-111/ SCD-121 (NCT03655678/ NCT03745287)	BCL11A enhancer	I/II	2	Transfusion independence	No off-target events; stable engraftment; no severe AEs	[62]
CLIMB THAL-111 (NCT03655678)	BCL11A enhancer	III	52	91% achieved transfusion independence	No deaths or malignancies; AEs similar to conditioning and HSCT	[94]
BRL-101 (NCT04211480)	BCL11A + 58 enhancer	I/II	6	100% transfusion independence	No drug-related deaths; most AEs from conditioning; transient thrombocytopenia resolved	[95]
CLIMB THAL-111/131 (NCT03655678/ NCT04208529)	BCL11A enhancer	III + follow-up	54	Sustained HRQoL improvement up to 48 months	No new safety concerns; benefits maintained over time	[96]

## Data Availability

Not applicable.
